# Matrix Effect of Diverse Biological Samples Extracted with Different Extraction Ratios on the Detection of β-*N*-Methylamino-L-Alanine by Two Common LC-MS/MS Analysis Methods

**DOI:** 10.3390/toxins14060387

**Published:** 2022-06-02

**Authors:** Peng Zhao, Jiangbing Qiu, Aifeng Li, Guowang Yan, Min Li, Ying Ji

**Affiliations:** 1 College of Environmental Science and Engineering, Ocean University of China, Qingdao 266100, China; zhaopeng4332@stu.ouc.edu.cn (P.Z.); qiujiangbing@ouc.edu.cn (J.Q.); yanguowang@stu.ouc.edu.cn (G.Y.); limin7668@stu.ouc.edu.cn (M.L.); jy@stu.ouc.edu.cn (Y.J.); 2 Key Laboratory of Marine Environment and Ecology, Ocean University of China, Ministry of Education, Qingdao 266100, China

**Keywords:** β-*N*-methylamino-L-alanine, extraction ratio, matrix effect, solid-phase extraction (SPE) purification, liquid chromatography-tandem mass spectrometry (LC-MS/MS)

## Abstract

Neurotoxin β-*N*-methylamino-L-alanine (BMAA) is hypothesized as an important pathogenic factor for neurodegenerative diseases such as amyotrophic lateral sclerosis/parkinsonism-dementia complex (ALS-PDC). Comparative study on the accuracy of BMAA analyzed by the regular LC-MS/MS methods is still limited for different biological matrices. In this study, a free-BMAA sample of cyanobacterium and BMAA-containing positive samples of diatom, mussel, scallop, and oyster were extracted with varied extraction ratios (ER) ranging from 1:20 to 1:2000. These extracts were then purified by MCX cartridges. After SPE purification, these different biological samples were analyzed by two common LC-MS/MS analysis methods, a direct analysis without derivatization by a hydrophilic interaction liquid chromatography (HILIC)-MS/MS and pre-column 6-aminoquinolyl-*N*-hydroxysuccinimidyl carbamate (AQC) derivatization combined with a C18 column. The results suggested that the recoveries of BMAA spiked in the cyanobacterial sample were close to 100% in the total soluble form extracts with the ER of 1:100 (g/mL) and the precipitated bound form extracts with the ER of 1:500. The recommended ER for the precipitated bound form of BMAA in diatoms and the total soluble form of BMAA in mollusks are 1:500 and 1:50, respectively. The quantitative results determined by the AQC derivatization method were lower than those determined by the direct analysis of the HILIC method in diatom and mollusk samples. The results of the HILIC method without the derivatization process were closer to the true value of BMAA in cyanobacteria. This work contributes to the performance of the solid-phase extraction (SPE) purification protocol and the accuracy of BMAA analysis by LC-MS/MS in diverse biological samples.

## 1. Introduction

The neurotoxic derivative of alanine β-*N*-methylamino-L-alanine (BMAA) can induce the death of neurons in the central nervous system [1], which seems to be an important pathogenic factor for neurodegenerative diseases such as amyotrophic lateral sclerosis/parkinsonism–dementia complex (ALS-PDC) [2]. BMAA is associated with protein misfolding and aggregation, the inhibition of specific enzymes, and neuroinflammation [3]. It is hypothesized that BMAA can be incorporated into protein synthesis as a substitute for alanine and serine [4]. The BMAA-containing proteins can also slowly release the free form of BMAA over time, which will cause chronic and adverse effects on neurons [5]. According to the source of BMAA, it was first discovered in the seeds of *Cycas circinalis* [6], and it was later inferred to be produced by the symbiotic *Nostoc* sp. of cycad root [7]. At present, BMAA is detected in some strains of cyanobacteria, diatoms, and dinoflagellate distributed worldwide in freshwater and marine ecosystems [8,9]. For example, the precipitated bound form of BMAA was detected in more than 20 diatom strains of the genera *Pseudo-nitzschia*, *Thalassiosira*, *Chaetoceros*, and *Planktoniella* isolated from the Chinese coast [8]. BMAA was also detected in diverse organisms, such as oysters, mussels, snails, and fish, at different trophic levels, which increases its exposure risk for humans [10,11,12,13]. In some previous studies, the accumulation of BMAA along food chains exhibited significant biomagnification behavior in marine ecosystems [12]. Therefore, it is necessary to strengthen the monitoring of BMAA in seafood products in order to protect human health.

So far, several isomers of BMAA, including 2,4-diamino butyric acid (DAB), β-amino-*N*-methylalanine (BAMA), and *N*-(2-aminoethyl) glycine (AEG), have been paid attention to during the analysis of BMAA in biological samples [14]. Meanwhile, three forms of BMAA were separated and analyzed in common analytical protocols for biological samples, including free form, soluble-bound form, and precipitated-bound form of BMAA. The free form of BMAA was extracted by trichloroacetic acid (TCA), the total soluble form of BMAA (containing both free and soluble-bound forms) was hydrolyzed by HCl from the supernatant extract of TCA, and the precipitated-bound form of BMAA was hydrolyzed by HCl from the centrifugal pellets of the extract [15]. Therefore, it is a challenge for analytical methods to effectively separate and correctly identify BMAA and its isomers from the complex matrix to avoid false-positive results [16]. In addition, since the content of BMAA is significantly lower than other amino acids in complex environmental samples, more sensitive methods are needed to analyze BMAA to avoid false-negative results [17]. The early analysis of BMAA was performed using an improved technique for amino acid analysis [18]. Gas chromatography-mass spectrometry (GC-MS) was also applied to analyze BMAA in samples of primates and cycad seeds [19,20]. Later, BMAA was detected by a high-performance liquid chromatography-fluorescence detector (HPLC-FLD) or HPLC-mass spectrometry (MS) using 6-aminoquinolyl-*N*-hydroxysccinimidyl carbamate (AQC) as a derivative [21,22]. Except for propyl chloroformate (PCF), ninhydrin, and dansyl chloride [23,24,25], AQC is the most commonly used derivative reagent for BMAA at present. Hydrophilic interaction liquid chromatography (HILIC) was also adopted to directly analyze BMAA without derivatization processes for toxin extract before analysis [26,27]. Currently, direct analysis with a HILIC column and derivatized reverse-phase LC column are the most used methods in the analytical works for BMAA [18]. However, both methods have some shortcomings in the analysis of natural samples. For example, different biological samples, especially the hydrolyzed extracts by HCl, have diverse amino acids that will influence the identification and quantification of BMAA, which needs technical expertise to improve its accuracy [15,28]. BMAA and its isomer BAMA could not be completely separated on some HILIC columns, which easily affects the quantitation of BMAA when much BAMA is present in samples, although BMAA can be quantified by the special transition *m*/*z* 119 -> 88 [29]. In addition, the interference of the sample matrix on the MS signal can lead to the underestimation or overestimation of analyte concentrations quantified by LC-MS/MS analysis [30]. To eliminate interfering compounds from the sample matrix, the clean-up steps of solid-phase extraction (SPE) were adopted during sample preparation [24,26,31,32]. Of course, the SPE purification protocol should be carefully optimized and carried out to achieve a satisfactory recovery [33]. So far, the knowledge of the matrix effect of different organisms, especially cyanobacteria and diatoms, on the LC-MS/MS analysis methods for BMAA is still limited.

In this study, diverse organisms, including cyanobacteria, diatoms, scallops, mussels, and oysters, were targeted, of which cyanobacteria were negative samples without BMAA, and the others were positive samples containing BMAA. These biological samples were extracted with different extraction ratios by hydrochloric acid of 20 mmol·L^−1^. The negative cyanobacterium samples were spiked with exogenous BMAA standard. All samples were then purified by MCX-cartridges and separately subjected to AQC derivatization and no derivatization. Based on the LC-MS/MS system, direct analysis without derivatization by a HILIC-MS/MS and a pre-column AQC derivatization combined with a C18 column were performed on the analysis of BMAA in phytoplankton and mollusk samples. The recoveries of BMAA on MCX-cartridges were evaluated in the extract of these samples with varied extraction ratios, and the analytical performance for both LC-MS/MS methods was compared. This work will further help us improve the extraction methods for different matrix samples in the LC-MS/MS analysis of BMAA.

## 2. Results and Discussion

### 2.1. Recovery of BMAA through SPE Cartridges for Different Sample Matrices

**Figure 1:**
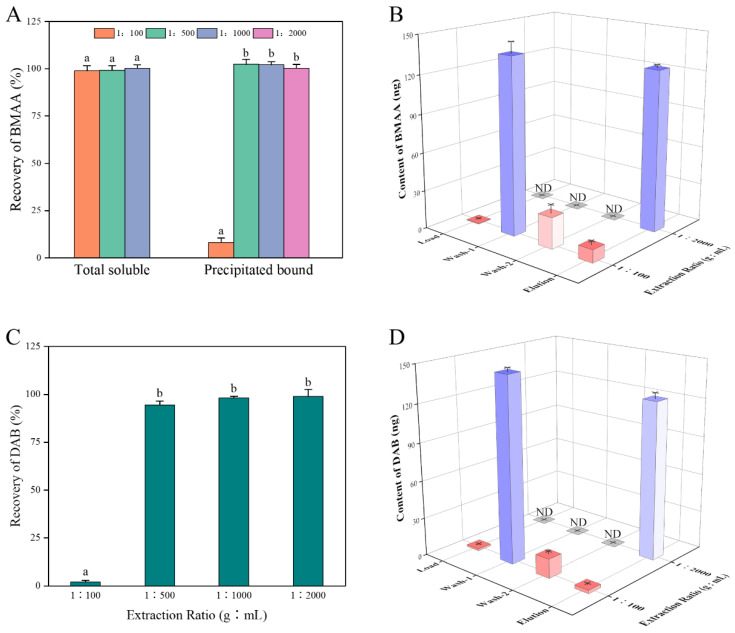
Recoveries (n = 3) of BMAA spiked in the total soluble, and the precipitated bound form extracts (**A**) and DAB spiked in the precipitated bound form extracts (**C**) at different extraction ratios of *Nostoc* sp.; collective contents (n = 3) in different SPE purification steps of BMAA (**B**) and DAB (**D**) spiked in the precipitated bound form extracts at different extraction ratios. Different letters (a, b) indicate significantly different values at *p* < 0.05.

The neurotoxin BMAA was not detected in the cyanobacterial sample of *Nostoc* sp. cultured in the laboratory in this study. The negative detection of BMAA is consistent with some previous studies for BMAA in cyanobacteria [26,27,34,35]. Recoveries of BMAA spiked in the cyanobacterial extracts with different extraction ratios on an Oasis-MCX SPE cartridge are shown in Figure 1. Results showed that the recoveries of BMAA were close to 100% in the total soluble extracts among the three groups with no significant difference, confirming the robust performance of the Oasis-MCX SPE cartridges to purify BMAA from the cyanobacterial matrix. Meanwhile, the recovery for precipitated bound BMAA was significantly lower in the extraction ratio of the 1:100 group than that in the other three experimental groups, which may be due to the presence of the more complex components in the precipitated bound extracts at the extract ratio at 1:100. Generally, the SPE purification efficiency decreases with the increase of the sample amount loaded on the cartridges [36]. Therefore, the appropriate extraction ratio is important for the SPE purification efficiency of BMAA from the cyanobacterial matrix. Individual solutions were collected separately for each of the 1:100 and 1:2000 experimental groups during SPE purification for further analysis of BMAA recovery in the precipitated bound extracts. In the case of the extraction ratio at 1:100, a very small amount of BMAA could not be held on the cartridges at the time of sample loading, while more than 90% of BMAA was detached from the cartridges during both rinsing processes, especially in the first washing step, resulting in a low recovery of 8% (Figure 1B). In the 1:2000 extraction ratio group, BMAA was eluted only in the final elution stage with a recovery of 95%. Only the precipitated bound form of the extract was simulated and spiked with DAB in this study due to no precipitated bound form of DAB reported before in cyanobacterial samples [37]. The pattern of DAB recovery was similar to those of BMAA in this study (Figure 1C,D). In the 1:100 extraction ratio group, a high proportion of DAB was washed out during the purification of the washing steps, resulting in a recovery of only 2%. The recovery of DAB reached up to between 94% and 99% in the other extraction ratio groups.

The strain of diatom *M. comicus* mainly produced the precipitated bound form of BMAA, while the total soluble form of DAB at 5.3 μg·g^−1^ was detected only at the extraction ratio of 1:100, and both the total soluble form of BMAA and precipitated bound form of DAB were absent (Table 1). The concentration of precipitated bound BMAA detected in the extraction ratio of the 1:100 group was significantly lower than that in the other two experimental groups. This result was mainly caused by the low recovery similar to that of cyanobacterial samples. No significant difference was present in the concentration of precipitated bound BMAA ranging from 4.1 to 4.2 μg·g^−1^ in the groups with the extraction ratios of 1:500 and 1:1000. The results suggest that an extraction ratio of 1:500 is recommended for the analysis of *M. comicus* with high recoveries and detectability. However, the extraction ratio of BMAA in microalgal samples was 1:100 in the previous study [35], which may lead to an underestimate of the precipitated bound BMAA. Based on the above results, the extraction ratios of 1:100 and 1:500 were recommended for the application of LC-MS/MS for the analysis of the total soluble and precipitated bound forms of BMAA, respectively, in cyanobacteria and diatoms.

**Figure 2:**
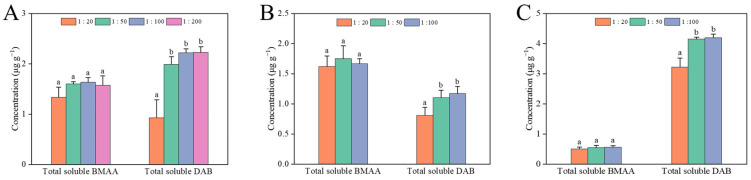
Quantitative results (n = 3) of the total soluble form BMAA and DAB in *Patinopecten yessoensis* (**A**), *Mytilus galloprovincialis* (**B**), and *Crassostrea gigas* (**C**) at different extraction ratios. Different letters (a, b) indicate significantly different values at *p* < 0.05.

The total soluble forms of BMAA and DAB were detected in the scallop, mussel, and oyster samples, which was consistent with the detection of shellfish in our previous study [10]. There was no significant difference in the concentrations of the total soluble BMAA in the shellfish extracts with different extraction ratios and the concentrations of the total soluble form of BMAA in scallops, mussels, and oysters were about 1.3, 1.7, and 0.6 μg·g^−1^, respectively (Figure 2). In some previous studies, the mean concentrations of BMAA in mussels and oysters from the west coast of Sweden were 0.18 and 0.06 μg·g^−1^, respectively [13], and that in mollusks from Taihu Lake, China, was 3.21 μg·g^−1^ [38]. In contrast, the mean concentrations of BMAA accumulated in bivalves, crustaceans, and gastropods were 0.84, 1.19, and 2.08 μg·g^−1^, respectively, in Jiaozhou Bay [8]. Therefore, the concentrations of BMAA in diverse shellfish were about several μg·g^−1^ levels. However, the concentration of the total soluble form of DAB in the shellfish extract with an extraction ratio of 1:20 was significantly lower than that in the other groups in all three shellfish, especially in scallops, and no obvious difference was found in the concentrations of the total soluble DAB when the extraction ratios were less than 1:20. In some previous studies, the extraction ratios of BMAA in shellfish samples were generally less than 1:50 [35,38,39]. Therefore, an optimized extraction ratio of 1:50 is recommended for the LC-MS/MS analysis of BMAA and its isomers in mollusk samples.

Some endogenous substances, such as proteins, lipids, or pigments, would be simultaneously extracted with the target compounds in the process of sample extraction, which can affect the ionization efficiency of the mass spectrometer in the LC-MS/MS system, resulting in an underestimate (signal suppression) or overestimate (signal enhancement) of quantitative results of the analyte [30]. The signal of BMAA was inhibited by about 10% in the total soluble extract of mussel tissues but significantly inhibited by about 65% after complete protein hydrolysis [40]. At the same time, BMAA showed a signal enhancement of about 18% in the cyanobacterial matrix [41]. In order to reduce the matrix effect on the analysis of BMAA, the SPE purification protocol was used in many studies to clean up samples. The Oasis-MCX SPE cartridges were widely used in the purification of the total soluble and precipitated bound forms of BMAA in some previous studies [10,26,35]. For example, the Oasis-MCX SPE cartridges were used to clean up extracts of cyanobacterial cultures to reduce the matrix effect in the analysis of BMAA [42]. The recoveries of BMAA and DAB in natural water were also investigated using different types of SPE cartridges, and finally the Oasis-MCX cartridge was found to be the best choice [33]. However, no optimized extraction ratios for different biological samples were reported in the analytical works for BMAA and its isomers using LC-MS/MS system before. To our knowledge, the present study is the first time to compare and optimize the purification efficiency of Oasis-MCX cartridges for BMAA and DAB in different biological samples with diverse extraction ratios.

### 2.2. Amino Acid Analysis

In order to explain the low recovery of BMAA through the SPE cartridges when higher extraction ratios were used in this study, the concentrations of common amino acids were monitored by LC-MS/MS in the total soluble and precipitated bound extracts of *Nostoc* sp. (Table 2). The Oasis-MCX SPE cartridges contain mixed-mode polymer sorbents characterized by reverse-phase and strong cationic functions that can selectively interact with neutral and basic compounds, of which the retention mechanisms for compounds include ion exchange and electrostatic interactions [36]. BMAA, as a non-protein amino acid, can be adsorbed and desorbed together with amino acids by MCX adsorbents. In the SPE purification process, amino acids can compete with BMAA and DAB for adsorption sites. A large number of amino acids would occupy more adsorption sites, resulting in a reduction of adsorption between BMAA and the adsorbent and the loss of some BMAA and DAB from the sample solution that cannot be fully retained on the column. In addition, even if BMAA and DAB are retained on the cartridge together with amino acids, due to the limited enrichment capacity of the adsorbent, the adsorption of excessive compounds would weaken the retention capacity of the adsorbent. Thus, BMAA and DAB may also be eluted during the rinsing process, resulting in low recoveries of BMAA and DAB. Previous studies have also shown that the recovery of BMAA decreased in the matrices containing many amino acids [43]. In the extracts with an extraction ratio of 1:100, the content of each amino acid in the precipitated bound extract was 4.9 to 620 times that in the total soluble extract, and the content ratios of total amino acids were about 20 between the precipitated bound form extracts (Table 2). Meanwhile, the recoveries of BMAA in the precipitated bound and total soluble form extracts were 8% and 98%, respectively. This discrepancy demonstrated that the higher content of amino acids caused the low recovery of BMAA through the SPE cartridges. The total contents of amino acids reached 14,050 ng·mL^−1^ in the cyanobacterial extract with the extraction ratio at 1:100, resulting in a decrease in the force between BMAA and SPE cartridge sorbent, and 80% of the BMAA was eluted in the first washing process. In the extract with the extraction ratio at 1:2000, the total content of amino acids was 703 ng·mL^−1^, and no significant loss of BMAA was observed during the SPE purification process, and its recovery reached up to 98%. In this study, the recovery of BMAA in the extract was 98% at an extraction ratio of 1:500, and its total content of amino acids was 2810 ng·mL^−1^. It is recommended that the total content of amino acids should be less than 2810 ng·mL^−1^ when using the Oasis-MCX (3 mL, 60 mg) cartridge for the purification and analysis of BMAA and its isomers. In addition, our previous study found that basic amino acids such as lysine, histidine, and arginine were eluted with BMAA during the SPE purification of MCX cartridges [42]. The elution time of these compounds was slightly later than that of DAB on the HILIC gradient. Although the detection of BMAA has no significant matrix effect, the detection of the precipitated bound form of DAB may be inhibited by the proximity of interfering compounds [42]. In this study, these amino acids were also found in the total soluble and precipitated bound extracts of cyanobacteria. At the extraction ratios of 1:500 or lower, the matrix effect of DAB was no more apparent with decreasing amino acid concentrations.

### 2.3. Comparison between Both Analytical Methods

**Figure 3:**
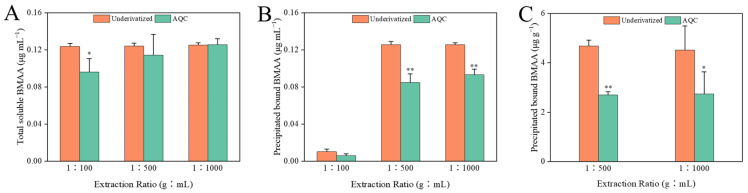
Differences in the total soluble form (**A**) and the precipitated bound form of BMAA (**B**) in cyanobacterium at different extraction ratios with two analytical methods (n = 3); differences in the precipitated bound form of BMAA (**C**) in diatom at different extraction ratios with both analytical methods (n = 3). * indicates *p* < 0.05; ** indicates *p* < 0.01.

**Figure 4:**
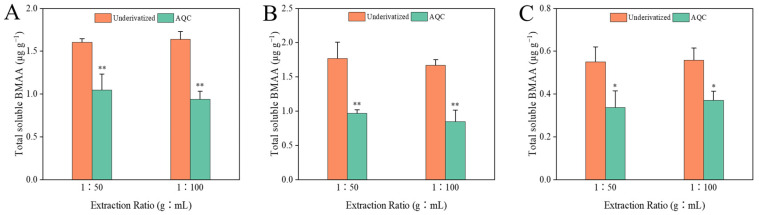
Differences in the total soluble form of BMAA in *Patinopecten yessoensis* (**A**), *Mytilus galloprovincialis* (**B**), and *Crassostrea gigas* (**C**) at different extraction ratios with both analytical methods (n = 3). * indicates *p* < 0.05; ** indicates *p* < 0.01.

The quantification of BMAA in the same purified samples was carried out separately by direct HILIC-MS/MS analysis and precolumn AQC-derivatization methods, ensuring that possible differences in the results were only related to the methods themselves and were not influenced by other factors. The LC-MS/MS chromatograms of the BMAA standard solution and the cyanobacterial samples analyzed by both methods are shown in Figure S1. The concentrations of the total soluble and precipitated bound forms of BMAA in the cyanobacterial samples with different extraction ratios determined by both methods are shown in Figure 3. In the total soluble extract with an extraction ratio of 1:100, the concentration of BMAA determined by the AQC derivatization method was about 78% of that determined by the HILIC method (Figure 3A), and the recoveries of total soluble BMAA were 78% and 99% for the AQC derivatization and HILIC methods, respectively. In the other two groups, no significant differences were found between the total soluble BMAA concentrations determined by both methods, with recoveries of 92~101% in both groups. In the precipitated bound extract with an extraction ratio of 1:100, no significant difference in the BMAA concentrations was found between the AQC derivatization and the HILIC methods, while the BMAA concentrations determined by the HILIC method were significantly higher (*p* < 0.01) than those measured by the AQC derivatization method in other two groups (Figure 3B). Similarly, the concentration of BMAA determined by the HILIC method was about 1.6~1.7 times that determined by the AQC derivatization method in both groups of precipitated bound extracts of diatom (Figure 3C). Only total soluble BMAA was detected in mollusk samples by both LC-MS/MS methods (Figure 4). The concentrations of BMAA determined by the HILIC method were approximately 1.6~1.9 times those determined by the AQC derivatization method in all shellfish groups. Based on the above data, the concentrations of BMAA determined by the HILIC method were 1.3~1.9 times higher than that determined by the AQC derivatization method, regardless of the type of sample and extracts.

In some previous studies, biased or false-positive results may occur in the analysis of BMAA by LC-FLD [16] because all amino acids and any compounds that react with fluorescent derivatization reagents can be detected by LC-FLD. For example, the commonly derivatized primary and secondary amines resulted in at least 50 compounds detected in acid-hydrolyzed cyanobacterial samples without purification by SPE [34]. Baker et al. (2018) reported that the detection results of cyanobacteria with the derivatized RPLC-MS/MS were significantly higher than those of HILIC-MS/MS [44], which was contrary to the results obtained in this study. The issue of low recovery of BMAA in cyanobacterial samples was raised in a single-laboratory validation of HILIC-MS/MS [29]. In the above cases, the samples were not purified by SPE and analyzed directly by HILIC-MS/MS. Therefore, the low recovery of BMAA in cyanobacterial samples could result from the matrix effects in the HILIC-MS/MS. In this study, the cyanobacterial samples with appropriate extraction ratios purified by SPE did not cause the problem of low recovery. Therefore, the detection result of HILIC was not lower than that of the AQC derivation method. On the contrary, the AQC derivatization method showed lower results for the determination of BMAA. Some previous studies suggested a possible matrix effect in the derivatized extracts of samples [41,45]. In addition, a partial loss of BMAA can occur during the derivatization process, resulting in a low recovery [15]. Therefore, given that the processes such as sample extraction and purification are identical, the results determined by the derivative method are more vulnerable to the influence of the sample matrix, resulting in an underestimate of results. In addition, the derivatization reagent added in this study was sufficient to complete the derivatization of all BMAA molecules in the sample. For example, in the cyanobacteria precipitated bound extract with an extraction ratio of 1:100, the amino acid and the spiked BMAA were fully derived by consuming approximately 2.2 nmol of AQC molecules, while the actual addition of AQC was 210 nmol. In terms of the accuracy of the test results, the BMAA content in the cyanobacterial samples analyzed by the HILIC method was closer to the spiked standard concentration (125 ng·mL^−1^, Figure 3), except for the precipitated bound extract with an extraction ratio of 1:100 due to a low recovery, while the BMAA contents measured by the AQC derivatization method were significantly lower than the spiked concentration in all cases, except for the total soluble extract groups with the extraction ratios of 1:500 and 1:1000. Therefore, the accuracy of the detection results of cyanobacteria by the HILIC method was generally higher than that of the AQC derivatization method. Unfortunately, the accuracy of both methods for analyzing positive samples such as diatoms and shellfish cannot be accurately assessed due to the lack of corresponding BMAA internal standards or blank samples.

## 3. Conclusions

In this study, the recoveries of BMAA through MCX-cartridges were evaluated in the cyanobacterium, diatom, scallop, mussel, and oyster samples with different extraction ratios, and two common LC-MS/MS methods for BMAA were compared. The recovery of BMAA can be affected by the sample matrix, especially the complex matrix, after acid hydrolysis. It is recommended to use extraction ratios of 1:100 and 1:500 for the total soluble and the precipitated bound forms of BMAA in phytoplankton samples, respectively, and an extraction ratio of 1:50 for the total soluble form of BMAA in mollusks to achieve high recoveries. The perfect performance was reconfirmed for the Oasis-MCX SPE cartridges in the analysis of different environmental samples. The concentration of BMAA determined by the HILIC method was generally higher than that determined by the AQC derivatization method regardless of the type of sample and extracts. Samples are recommended to be purified with an MCX-cartridge before the analysis of BMAA and its isomers by the non-derivatization HILIC method.

## 4. Materials and Methods

### 4.1. Chemicals and Reagents

The reference materials of L-BMAA hydrochloride and DL-2,4-diaminobutyric acid dihydrochloride (DAB) were purchased from Sigma-Aldrich (Oakville, ON, Canada), and *N*-2 (aminoethyl) glycine (AEG, A608975) standard was purchased from Toronto Research Chemicals Inc. (Toronto, ON, Canada). Acetonitrile, formic acid, and methanol came from Merck KGaA (Darmstadt, Germany) and were chromatographic grades. Ammonium formate (NH_4_COOH) and trichloroacetic acid (TCA) were obtained from Sigma-Aldrich. The AccQ-Fluor Reagent Kit was obtained from the Waters (Taunton, MA, USA). Ultrapure water (resistivity > 18.2 MΩ·cm) was supplied by the MilliQ Water purification System (Millipore Ltd., SAS. Mosheim, France).

### 4.2. Preparation of Biological Samples

The cyanobacterial strain of *Nostoc* sp. was purchased from the freshwater algae collection in the Institute of Hydrobiology (FACHB), Chinese Academy of Sciences, and cultured using a BG11 medium. The diatom of *Minidiscus comicus* came from the South China Normal University diatom collection and was cultured in sterile-filtered (0.45 μm membrane, Xingya Ltd., Shanghai, China) seawater before enrichment with f/2 medium. The two strains were kept at 20 °C and 100 μmol·m^−2^·s^−1^ with a 12 h light/12 h dark cycle. Cells of both phytoplankton at the stationary growth phase were collected by centrifugation at 6200× *g* for 5 min. The biomass pellets were frozen at −80 °C for 12 h and lyophilized for 12 h in a vacuum freeze dryer, then ground into homogeneous powder by a multi-sample tissue grinder (Tissuelyser-48, Shanghai Jingxin Industrial Development Co., Ltd., Shanghai, China). Positive shellfish (containing BMAA) of scallop (*Mizuhopecten yessoensis*), mussel (*Mytilus galloprovincialis*), and oyster (*Magallana gigas*) were obtained from the seafood markets in Dalian, Huludao and Tianjin cities, respectively. All shellfish were dissected, and the soft tissues were homogenized and collected.

### 4.3. Extraction and Purification of Samples

#### 4.3.1. Extraction for The Total Soluble form of BMAA

A subsample of the 30 mg homogenized phytoplankton sample was mixed with 3 mL of 0.1 mol·L^−1^ TCA and transferred to a 10 mL centrifuge tube. The mixture was freeze–thawed in liquid nitrogen three times for 10 min each. Samples were sonicated in an ice bath for 5 min with a sonication probe (KS-750F, Kesheng Ultrasonic Equipment Ltd., Ningbo City, China) and were centrifuged at 6576× *g* for 10 min at 4 °C. Then, 2 mL of supernatant were taken and dried in nitrogen gas at 55 °C. The residual material was redissolved in 6 mol·L^−1^ HCl and hydrolyzed at 110 °C for 24 h to obtain the soluble-bound form of BMAA. The hydrolyzed solution was also dried in nitrogen gas at 55 °C and reconstituted with 1 mL of 20 mmol·L^−1^ HCl. The cyanobacterial extracts were diluted to 2, 10, and 20 times with 20 mmol·L^−1^ of HCl, corresponding to the extraction ratios (dry weight, g/mL) at 1:100, 1:500, and 1:1000, respectively. Since cyanobacterial extracts do not contain BMAA, the BMAA standard was added to the diluted extracts to make a concentration of 125 ng·mL^−1^ before the SPE purification. The diatom extracts were diluted 2, 4, and 10 times corresponding to the extraction ratios at 1:100, 1:200, and 1:500, respectively. The strain of *Minidiscus comicus* was positive for BMAA; thus, no exogenous BMAA was added to the diatom extracts. Each sample was prepared in triplicate.

Homogenized shellfish tissues of 250 mg were mixed with 2 mL of 0.1 mol·L^−1^ TCA and ground by a multi-sample tissue grinder. The supernatant was collected by centrifugation at 6576× *g* for 10 min at 4 °C. The pellet was extracted twice with an additional 1 mL of 0.1 mol·L^−1^ TCA, and all extracts were combined in the volumetric flask and made up to 5 mL with 0.1 mol·L^−1^ TCA. Two mL of the subsample were dried, hydrolyzed, and dissolved as described above. The extraction ratios (wet weight, g/mL) of shellfish extracts were 1:20, 1:50, 1:100, and 1:200, respectively. Each sample was prepared in triplicate. The mollusk samples were also positive, and no exogenous BMAA was added.

#### 4.3.2. Extraction of Precipitated Bound BMAA

During the extraction of total soluble BMAA, the centrifugal pellets were collected for the analysis of precipitated bound BMAA. The pellet was mixed with 6 mol·L^−1^ HCl and transferred to a 4 mL glass bottle. The mixture was hydrolyzed at 110 °C for 24 h and followed by centrifugation at 6577× *g* for 10 min. Two mL of supernatant were dried with nitrogen gas and reconstituted in 1 mL of 20 mmol·L^−1^ HCl. The extracts of phytoplankton and shellfish were diluted to the extraction ratios of 1:100, 1:500, 1:1000, 1:2000, 1:20, 1:50, 1:100, and 1:200, respectively. Each sample was prepared in triplicate.

#### 4.3.3. Purification of Extracts

The extracts were purified by Oasis-MCX (3 cc, 60 mg, Waters) cartridges referring to the method in our previous study with a minor modification [42]. The cartridge was activated by 3 mL of 100% methanol and equilibrated by 2 mL of ultrapure water. Then, 1 mL of extracts was loaded, and the cartridge was washed with 2 mL of 0.1 mol·L^−1^ HCl and 2 mL of 100% methanol successively and eluted with 3 mL of 5% NH_4_OH. The solutions eluted from each stage were collected separately and dried under nitrogen gas at 55 °C. The residue was redissolved in 1 mL of 20 mmol·L^−1^ HCl, filtered through a 0.22 μm polyether sulfone membrane to a vial, and stored at −20 °C for LC-MS/MS analysis.

### 4.4. Analytical Methods for BMAA

#### 4.4.1. HILIC-MS/MS Analysis of BMAA

Samples were analyzed using a Thermo Ultimate 3000 HPLC (Thermo Fisher Scientific, Bremen, Germany) in tandem with an AB Sciex Qtrap 4500 mass spectrometer (AB Sciex Pte. Ltd., Singapore) combined with an electrospray ionization source. The parameters of both the LC and MS systems were the same as those described in our previous study [8]. A SeQuant^®^ ZIC-HILIC column (150 mm × 2.1 mm, 5 µm) was used to separate BMAA using a binary mobile phase. Solvent A was water containing 0.1% formic acid, and solvent B was acetonitrile containing 0.1% formic acid. The injection volume was 5 µL. The gradient was run at 350 μL·min^−1^ at 30 °C from 95% to 60% B over 19 min, decreased to 40% B at 25 min, increased to 95% B at 27.01 min, and held for 2.99 min before re-equilibration for the next run. Mass spectrometry parameters were set as follows: curtain gas pressure of 40 psi, spray voltage of 5500 V, source temperature of 350 °C, source gas 1 and 2 at a pressure of 55 psi and 50 psi, respectively, and collision cell entrance potential of 10 V. The selective reaction monitoring (SRM) mode with five transitions (*m*/*z* 119 ->102, 101, 88, 56, and 44) was used to quantify BMAA and its isomers (Table S1). The limits of detection of BMAA and DAB were 3.4 and 0.65 ng·mL^−1^, respectively.

#### 4.4.2. LC-MS/MS Analysis with Precolumn AQC Derivatization

In order to compare both BMAA analysis methods, the samples were analyzed again using the AQC derivatization method on the same LC-MS/MS system [8]. The purified extracts were derived using an AccQ-Fluor Reagent Kit, and the derivatization operation followed the guide of the instruction manual. A Phenomenex Kinetex column (100 × 2.1 mm, 1.7 µm, C18, 100 Å) was used to separate AQC-derivatives of BMAA and other amino acids at 65 °C using a binary mobile phase. Solvent A was water containing 20 mmol·L^−1^ ammonium formate, and solvent B was methanol. The injection volume was 5 µL. The gradient was run at 350 μL·min^−1^ from 28% to 38% B over 2.5 min, held for 1 min with 38% B, increased to 85% B at 4 min, increased to 90% B at 4.25 min, held for 1 min with 90% B, decreased to 28% B at 5.45 min, and held for 1.55 min before re-equilibration for the next run. Mass spectrometry parameters were set as follows: curtain gas pressure of 40 psi, spray voltage of 5500 V, source temperature of 325 °C, source gas 1 and 2 at a pressure of 50 psi and 60 psi, respectively, and collision cell entrance potential of 10 V. Six transitions (*m*/*z* 459 ->119, 188, 214, 258, 289, and 171) were used for BMAA and its isomers (Table S1). The limits of detection of BMAA and DAB were 2.8 and 1.7 ng·mL^−1^, respectively.

### 4.5. LC-MS/MS Analysis of Amino Acids

A Phenomenex Kinetex column (100 × 2.1 mm, 1.7 µm, C18, 100 Å) was used to separate amino acids with a binary mobile phase. Solvent A was water containing 2.0 mmol·L^−1^ formic acid and 50 mmol·L^−1^ ammonium formate, and the solvent B was methanol. The injection volume was 5 µL. The gradient was run at 300 μL min^−1^ at 30 °C from 90% to 60% B over 3 min, held for 10 min with 60% B, decreased to 40% B at 18 min, held for 9 min with 40% B, increased to 90% B at 29 min, and held for 1 min before re-equilibration for the next sample. Mass spectrometry parameters were set as follows: curtain gas pressure of 40 psi, spray voltage of 5500 V, source temperature of 450 °C, source gas 1 and 2 at a pressure of 55 psi and 55 psi, respectively, and collision cell entrance potential of 10 V. All MRM transitions used for detection of amino acids are shown in Table S2.

### 4.6. Data Processing

All data and figures were processed using the Office Excel version 2019 software and Origin 2019 software. The SPSS statistical software package Version 25 was used for statistical analysis, and significant differences were identified by one-way analysis of variance (ANOVA). Different letters indicated significantly different values when *p* < 0.05 or 0.01 (ANOVA).

## Figures and Tables

**Table 1:** Concentration (μg·g^−1^, mean ± standard deviation, n = 3) of the total soluble and the precipitated bound forms of BMAA and DAB in diatom of *Minidiscus comicus* analyzed by HILIC-MS/MS.

ER ^#^	Total Soluble Form			Precipitated Bound Form		
	1:100	1:200	1:500	1:100	1:500	1:1000
BMAA	LOD	ND	ND	1.2 ± 0.24 ^a^	4.7 ± 0.24 ^b^	4.5 ± 0.97 ^b^
DAB	0.54 ± 0.03	LOD	LOD	ND	ND	ND

Note: # ER means extraction ratio of sample weight g to final extract volume mL. LOD: below the limit of detection; ND: not detected. a and b letters indicate *p* < 0.05.

**Table 2:** The concentrations (ng·mL^−1^, mean ± standard deviation, n = 3) of amino acids in the total soluble form and the precipitated bound form extracts of *Nostoc* sp.

ER ^#^	Total Soluble Form Extracts	Precipitated Bound Form Extracts		
	1:100	1:100	1:500	1:2000
Glycine	35 ± 2.7	612 ± 52	122 ± 10	30 ± 2.5
Alanine	58 ± 2.0	1462 ± 199	282 ± 40	70 ± 10
Serine	20 ± 3.8	371 ± 38	74 ± 7.6	18 ± 1.9
Proline	32 ± 1.7	662 ± 50	132 ± 10	33 ± 2.5
Valine	50 ± 4.6	1282 ± 193	256 ± 10	64 ± 2.5
Threonine	30 ± 1.5	688 ± 82	138 ± 16	34 ± 4.0
Isoleucine	44 ± 1.7	1224 ± 98	245 ± 20	61 ± 5.0
Leucine	54 ± 2.2	1385 ± 70	277 ± 14	69 ± 3.5
Aspartic acid	41 ± 0.49	199 ± 29	40 ± 5.8	10 ± 1.4
Glutamine	85 ± 8.6	1704 ± 136	341 ± 27	85 ± 6.8
Lysine	79 ± 7.8	1605 ± 169	321 ± 34	80 ± 8.5
Glutamic	9.0 ± 0.28	95 ± 9.9	19 ± 2.0	4.8 ± 0.49
Methionine	0.50 ± 0.38	310 ± 16	62 ± 3.2	16 ± 0.79
Histidine	5.0 ± 1.2	108 ± 1.2	22 ± 0.24	5.5 ± 0.060
Phenylalanine	62 ± 1.2	1402 ± 80	280 ± 16	70 ± 4.0
Arginine	88 ± 5.8	830 ± 64	166 ± 13	42 ± 3.2
Tyrosine	2.6 ± 0.58	109 ± 16	22 ± 3.2	5.5 ± 0.81
Tryptophan	0.089 ± 0.011	2.3 ± 0.0076	0.46 ± 0.0015	0.12 ± 0.00038
Total	694 ± 40	14047 ± 1348	2810 ± 270	702 ± 62

Note: # ER means extraction ratio of sample weight g to final extract volume mL.

## Data Availability

The data presented in this study are available in this article and supplementary material.

## Supplementary Materials

The following supporting information can be downloaded at: https://www.mdpi.com/article/10.3390/toxins14060387/s1, Figure S1: The LC-MS/MS chromatograms of BMAA, DAB and AEG standard detected by the HILIC direct analysis (A) and AQC derivatization method (B); Table S1: The MRM transitions used for detection of BMAA and its isomers; Table S2: The MRM transitions used for detection of amino acids.

## References

[1] Soto T., Buzzi E.D., Rotstein N.P., German O.L., Politi L.E. (2020). Damaging Effects of BMAA on Retina Neurons and Müller Glial Cells. Exp. Eye Res..

[2] Spencer P.S., Nunn P.B., Hugon J., Ludolph A.C., Ross S.M., Roy D.N., Robertson R.C. (1987). Guam Amyotrophic Lateral Sclerosis-Parkinsonism Dementia Linked to a Plant Excitant Neurotoxin. Science.

[3] Nunes-Costa D., Magalhães J.D., G-Fernandes M., Cardoso S.M., Empadinhas N. (2020). Microbial BMAA and the Pathway for Parkinson’s Disease Neurodegeneration. Front. Aging Neurosci..

[4] Glover W.B., Mash D.C., Murch S.J. (2014). The Natural Non-Protein Amino Acid *N*-β-Methylamino-L-Alanine (BMAA) Is Incorporated into Protein during Synthesis. Amino Acids.

[5] Murch S.J., Cox P.A., Banack S.A. (2004). A Mechanism for Slow Release of Biomagnified Cyanobacterial Neurotoxins and Neurodegenerative Disease in Guam. Proc. Natl. Acad. Sci. USA.

[6] Vega A., Bell E.A. (1967). α-Amino-β-Methylaminopropionic Acid, a New Amino Acid from Seeds of *Cycas circinalis*. Phytochemistry.

[7] Cox P.A., Banack S.A., Murch S.J., Rasmussen U., Tien G., Bidigare R.R., Metcalf J.S., Morrison L.F., Codd G.A., Bergman B. (2005). Diverse Taxa of Cyanobacteria Produce β-*N*-Methylamino-L-Alanine, a Neurotoxic Amino Acid. Proc. Natl. Acad. Sci. USA.

[8] Wang C., Yan C., Qiu J., Liu C., Yan Y., Ji Y., Wang G., Chen H., Li Y., Li A. (2021). Food Web Biomagnification of the Neurotoxin β-*N*-Methylamino-L-Alanine in a Diatom-Dominated Marine Ecosystem in China. J. Hazard. Mater..

[9] Jiang L., Eriksson J., Lage S., Jonasson S., Shams S., Mehine M., Ilag L.L., Rasmussen U. (2014). Diatoms: A Novel Source for the Neurotoxin BMAA in Aquatic Environments. PLoS ONE.

[10] Li A., Hu Y., Song J., Wang S., Deng L. (2018). Ubiquity of the Neurotoxin β-*N*-Methylamino-L-Alanine and Its Isomers Confirmed by Two Different Mass Spectrometric Methods in Diverse Marine Mollusks. Toxicon.

[11] Main B.J., Bowling L.C., Padula M.P., Bishop D.P., Mitrovic S.M., Guillemin G.J., Rodgers K.J. (2018). Detection of the Suspected Neurotoxin β-Methylamino-L-Alanine (BMAA) in Cyanobacterial Blooms from Multiple Water Bodies in Eastern Australia. Harmful Algae.

[12] Réveillon D., Abadie E., Séchet V., Masseret E., Hess P., Amzil Z. (2015). β-*N*-Methylamino-L-Alanine (BMAA) and Isomers: Distribution in Different Food Web Compartments of Thau Lagoon, French Mediterranean Sea. Mar. Environ. Res..

[13] Jonasson S., Eriksson J., Berntzon L., Spáčcil Z., Ilag L.L., Ronnevi L.O., Rasmussen U., Bergman B. (2010). Transfer of a Cyanobacterial Neurotoxin within a Temperate Aquatic Ecosystem Suggests Pathways for Human Exposure. Proc. Natl. Acad. Sci. USA.

[14] Jiang L., Aigret B., De Borggraeve W.M., Spacil Z., Ilag L.L. (2012). Selective LC-MS/MS Method for the Identification of BMAA from Its Isomers in Biological Samples. Anal. Bioanal. Chem..

[15] Faassen E.J., Antoniou M.G., Beekman-Lukassen W., Blahova L., Chernova E., Christophoridis C., Combes A., Edwards C., Fastner J., Harmsen J. (2016). A Collaborative Evaluation of LC-MS/MS Based Methods for BMAA Analysis: Soluble Bound BMAA Found to Be an Important Fraction. Mar. Drugs.

[16] Faassen E.J., Gillissen F., Lürling M. (2012). A Comparative Study on Three Analytical Methods for the Determination of the Neurotoxin BMAA in Cyanobacteria. PLoS ONE.

[17] Banack S.A., Murch S.J. (2018). Methods for the Chemical Analysis of β-*N*-Methylamino-L-A Lanine: What Is Known and What Remains to Be Determined. Neurotox. Res..

[18] Bishop S.L., Murch S.J. (2020). A Systematic Review of Analytical Methods for the Detection and Quantification of β-*N*-Methylamino-L-Alanine (BMAA). Analyst.

[19] Duncan M.W., Kopin I.J., Crowley J.S., Jones S.M., Markey S.P. (1989). Quantification of the Putative Neurotoxin 2-Amino-3-(Methylamino)Propanoic Acid (Bmaa) in Cycadales: Analysis of the Seeds of Some Members of the Family Cycadaceae. J. Anal. Toxicol..

[20] Duncan M.W., Markey S.P., Weick B.G., Pearson P.G., Ziffer H., Hu Y., Kopin I.J. (1992). 2-Amino-3-(Methylamino)Propanoic Acid (BMAA) Bioavailability in the Primate. Neurobiol. Aging.

[21] Cox P.A., Banack S.A., Murch S.J. (2003). Biomagnification of Cyanobacterial Neurotoxins and Neurodegenerative Disease among the Chamorro People of Guam. Proc. Natl. Acad. Sci. USA.

[22] Banack S.A., Cox P.A. (2003). Biomagnification of Cycad Neurotoxins in Flying Foxes: Implications for ALS-PDC in Guam. Neurology.

[23] Banack S.A., Johnson H.E., Cheng R., Alan Cox P. (2007). Production of the Neurotoxin BMAA by a Marine Cyanobacterium. Mar. Drugs.

[24] Esterhuizen-Londt M., Downing S., Downing T.G. (2011). Improved Sensitivity Using Liquid Chromatography Mass Spectrometry (LC-MS) for Detection of Propyl Chloroformate Derivatised β-*N*-Methylamino-L-Alanine (BMAA) in Cyanobacteria. Water SA.

[25] Lampinen Salomonsson M., Hansson A., Bondesson U. (2013). Development and In-House Validation of a Method for Quantification of BMAA in Mussels Using Dansyl Chloride Derivatization and Ultra Performance Liquid Chromatography Tandem Mass Spectrometry. Anal. Methods.

[26] Kubo T., Kato N., Hosoya K., Kaya K. (2008). Effective Determination Method for a Cyanobacterial Neurotoxin, β-*N*-Methylamino-L-Alanine. Toxicon.

[27] Rosén J., Hellenäs K.E. (2008). Determination of the Neurotoxin BMAA (β-*N*-Methylamino-L-Alanine) in Cycad Seed and Cyanobacteria by LC-MS/MS (Liquid Chromatography Tandem Mass Spectrometry). Analyst.

[28] Faassen E.J. (2014). Presence of the Neurotoxin BMAA in Aquatic Ecosystems: What Do We Really Know?. Toxins.

[29] Tymm F.J.M., Bishop S.L., Murch S.J. (2021). A Single Laboratory Validation for the Analysis of Underivatized β-*N*-Methylamino-L-Alanine (BMAA). Neurotox. Res..

[30] Taylor P.J. (2005). Matrix Effects: The Achilles Heel of Quantitative High-Performance Liquid Chromatography-Electrospray-Tandem Mass Spectrometry. Clin. Biochem..

[31] Roney B.R., Renhui L., Banack S.A., Murch S., Honegger R., Cox P.A. (2009). Consumption of Fa Cai Nostoc Soup: A Potential for BMAA Exposure from Nostoc Cyanobacteria in China?. Amyotroph. Lateral Scler..

[32] Spáil Z., Eriksson J., Jonasson S., Rasmussen U., Ilag L.L., Bergman B. (2010). Analytical Protocol for Identification of BMAA and DAB in Biological Samples. Analyst.

[33] Yan B., Liu Z., Huang R., Xu Y., Liu D., Lin T.F., Cui F. (2017). Optimization of the Determination Method for Dissolved Cyanobacterial Toxin BMAA in Natural Water. Anal. Chem..

[34] Krüger T., Mönch B., Oppenhäuser S., Luckas B. (2010). LC-MS/MS Determination of the Isomeric Neurotoxins BMAA (β-*N*-Methylamino-L-Alanine) and DAB (2,4-Diaminobutyric Acid) in Cyanobacteria and Seeds of *Cycas revoluta* and *Lathyrus latifolius*. Toxicon.

[35] Réveillon D., Abadie E., Séchet V., Brient L., Savar V., Bardouil M., Hess P., Amzil Z. (2014). Beta-*N*-Methylamino-L-Alanine: LC-MS/MS Optimization, Screening of Cyanobacterial Strains and Occurrence in Shellfish from Thau, a French Mediterranean Lagoon. Mar. Drugs.

[36] Aparicio-Muriana M.M., Carmona-Molero R., Lara F.J., García-Campaña A.M., del Olmo-Iruela M. (2022). Multiclass Cyanotoxin Analysis in Reservoir Waters: Tandem Solid-Phase Extraction Followed by Zwitterionic Hydrophilic Interaction Liquid Chromatography-Mass Spectrometry. Talanta.

[37] Faassen E.J., Gillissen F., Zweers H.A.J., Lrling M. (2009). Determination of the Neurotoxins BMAA (β-*N*-Methylamino-L-Alanine) and DAB (α-,γ-Diaminobutyric Acid) by LC-MSMS in Dutch Urban Waters with Cyanobacterial Blooms. Amyotroph. Lateral Scler..

[38] Jiao Y., Chen Q., Chen X., Wang X., Liao X., Jiang L., Wu J., Yang L. (2014). Occurrence and Transfer of a Cyanobacterial Neurotoxin β-Methylamino-L-Alanine within the Aquatic Food Webs of Gonghu Bay (Lake Taihu, China) to Evaluate the Potential Human Health Risk. Sci. Total Environ..

[39] Lepoutre A., Faassen E.J., Zweers A.J., Lürling M., Ge A., Lance E. (2020). How the Neurotoxin β-*N*-Methylamino-L-Alanine Accumulates in Bivalves: Distribution of the Different Accumulation Fractions among Organs. Toxins.

[40] Beach D.G., Kerrin E.S., Giddings S.D., Quilliam M.A., McCarron P. (2018). Differential Mobility-Mass Spectrometry Double Spike Isotope Dilution Study of Release of β-Methylaminoalanine and Proteinogenic Amino Acids during Biological Sample Hydrolysis. Sci. Rep..

[41] Jiang L., Johnston E., Åberg K.M., Nilsson U., Ilag L.L. (2013). Strategy for Quantifying Trace Levels of BMAA in Cyanobacteria by LC/MS/MS. Anal. Bioanal. Chem..

[42] Li A., Fan H., Ma F., McCarron P., Thomas K., Tang X., Quilliam M.A. (2012). Elucidation of Matrix Effects and Performance of Solid-Phase Extraction for LC-MS/MS Analysis of β-*N*-Methylamino-l-Alanine (BMAA) and 2,4-Diaminobutyric Acid (DAB) Neurotoxins in Cyanobacteria. Analyst.

[43] Esterhuizen-Londt M., Downing T.G. (2011). Solid Phase Extraction of β-*N*-Methylamino-L-Alanine (BMAA) from South African Water Supplies. Water SA.

[44] Baker T.C., Tymm F.J.M., Murch S.J. (2018). Assessing Environmental Exposure to β-*N*-Methylamino-L-Alanine (BMAA) in Complex Sample Matrices: A Comparison of the Three Most Popular LC-MS/MS Methods. Neurotox. Res..

[45] Lage S., Burian A., Rasmussen U., Costa P.R., Annadotter H., Godhe A., Rydberg S. (2016). BMAA Extraction of Cyanobacteria Samples: Which Method to Choose?. Environ. Sci. Pollut. Res..

