# Supplementary material for: Matrix Effect of Diverse Biological Samples Extracted with Different Extraction Ratios on the Detection of β-N-Methylamino-L-Alanine by Two Common LC-MS/MS Analysis Methods
Source: Toxins (Basel). 2022 Jun 2;14(6):387. doi: 10.3390/toxins14060387 (PMC9230712; doi:10.3390/toxins14060387)
Supplement: Supplementary file 1 [file toxins-14-00387-s001.zip › toxins-1738060-supplementary.pdf]

Supplementary Materials: Matrix Effect of Diverse Biological Samples Extracted with Different Extraction Ratios on the Detection of β-*N*-methylamino-L-alanine by Two Common LC-MS/MS Analysis Methods

Peng Zhao, Jiangbing Qiu, Aifeng Li, Guowang Yan, Min Li and Ying Ji

Table S1. The MRM transitions used for detection of BMAA and its isomers.

Methods	Precursor ion (*m*/*z*)	Product ion (*m*/*z*)	DP (V)	CE (eV)
**HILIC**	119	102	30	13
	119	101	30	11
	119	88	30	17
	119	56	30	24
	119	44	30	24
**AQC**	459	119	30	25
	459	188	30	25
	459	214	30	40
	459	258	30	30
	459	289	30	25
	459	171	30	40

Table S2. The MRM transitions used for detection of amino acids.

Methods	Precursor ion (*m*/*z*)	Product ion (*m*/*z*)	DP (V)	CE (eV)
**Glycine**	76	30	20	10
	117	76	20	10
**Alanine**	90	44	40	15
	90	45	20	45
**Serine**	106	60	40	15
	106	42	40	30
**Proline**	116	70	40	20
	157	70	20	40
**Valine**	118	72	40	20
	118	55	40	30
**Threonine**	120	74	40	15
	120	56	40	25
**Cysteine**	122	59	40	35
	163	122	20	10
**Isoleucine**	132	69	40	30
	132	86	40	15
**Leucine**	132	86	40	15
	132	44	40	35
**Asparagine**	133	87	30	12
	133	74	30	16
**Aspartic acid**	134	74	30	15

	134	88	30	18
**Glutamine**	147	84	40	20
	147	130	40	15
**Lysine**	147	67	40	14
	147	84	40	30
**Glutamic amid**	148	84	30	16
	148	56	30	28
**Methionine**	150	61	40	35
	150	56	40	20
**Histidine**	156	110	40	20
	156	82	40	45
**Phenylalanine**	166	120	40	15
	166	103	40	40
**Arginine**	175	70	20	35
	175	60	20	15
**Tyrosine**	182	91	40	25
	182	136	40	30
**Tryptophan**	205	146	40	20
	205	118	40	35

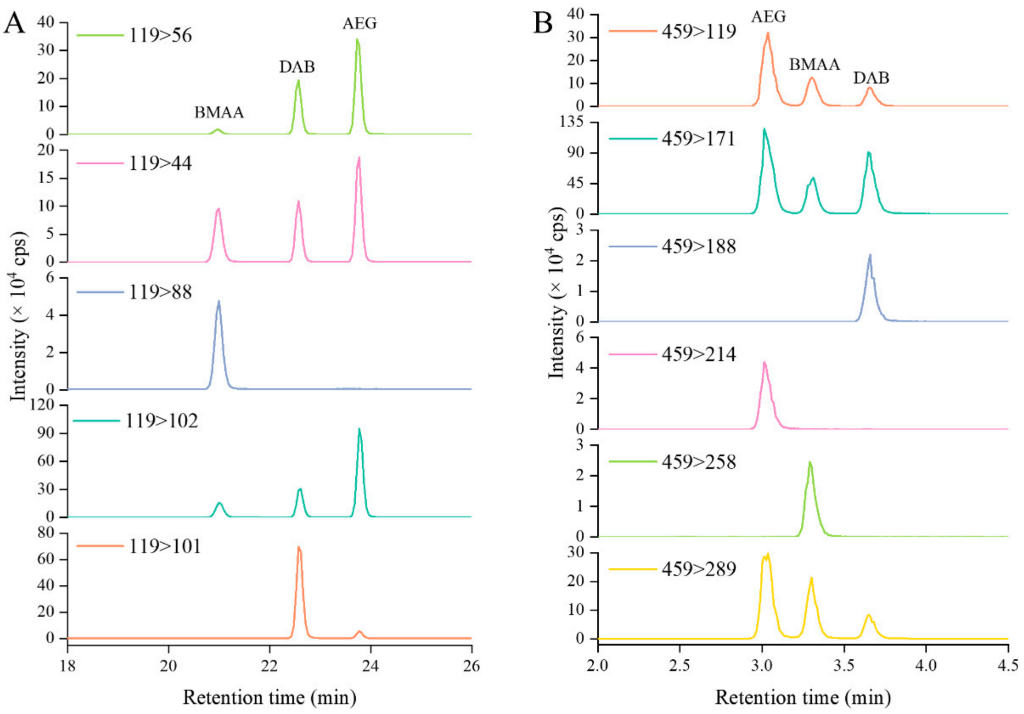

Figure S1. The LC-MS/MS chromatograms of BMAA, DAB and AEG standard detected by the HILIC direct analysis (A) and AQC derivatization method (B).
